# FOXP3 variants are independently associated with transforming growth factor Β1 plasma levels in female patients with inflammatory bowel disease

**DOI:** 10.1016/j.clinsp.2022.100084

**Published:** 2022-07-27

**Authors:** Cláudia Junko Inoue, Tamires Flauzino, Beatriz Piantoni Gonçalves, Jaqueline Costa Castardo de Paula, Talita Cristina Galvão, Paula Kikuchi Miyazaki, Camila Cataldi de Alcantara, Lucilene Rosa e Silva Westmore, Marcell Alysson Batisti Lozovoy, Edna Maria Vissoci Reiche, Andréa Name Colado Simão

**Affiliations:** a Laboratory of Research in Applied Immunology, Universidade Estadual de Londrina, Londrina, PR, Brazil; b Outpatient Clinic of Gastroenterology, Hospital Universitário, Universidade Estadual de Londrina, Londrina, PR, Brazil; c Department of Pathology, Clinical Analysis and Toxicology, Health Sciences Center, Universidade Estadual de Londrina, Londrina, PR, Brazil

**Keywords:** Inflammatory bowel diseases, Ulcerative colitis, Crohn's disease, Forkhead box protein 3 genetic variants, Transforming growth Factor β1

## Abstract

**Objective:**

The aim of this study was to evaluate the association of -924 G>A (rs2232365) and -3279 C>A (rs3761548) FOXP3 variants with IBD susceptibility, clinical and endoscopic activity, and IL-10 and TGF-β1 plasma levels.

**Method:**

The study included 110 IBD female patients, 60 with Ulcerative Colitis (UC) and 50 with Crohn's Disease (CD), and 154 female controls. FOXP3 variants were determined with Polymerase Chain Reaction-Restriction Fragment Length Polymorphism (PCR-RFLP). Plasma levels of IL-10 and TGF-β1 were determined using immunofluorimetric assay.

**Results:**

AA genotype of rs2232365 and rs3761548 was associated with CD (OR = 3.147, 95% CI 1.015–9.758, p = 0.047) and UC (OR = 3.221, 95% CI 1.050–9.876, p = 0.041) susceptibility, respectively. However, were not associated with TGF-β1 and IL-10 levels, and endoscopic/clinical activity disease. GAGA haplotype was associated with IBD (OR = 4.003, 95% CI 1.100–14.56, p = 0.035) and UC susceptibility (OR = 6.107, 95% CI 1.609–23.18, p = 0.008). In addition, IBD patients with the GAGA haplotype had lower TGF-β1 levels (p = 0.041). Moreover, G/C haplotype (dominant model) had a protective effect of 60% in CD susceptibility and lower Endoscopic Severity Index.

**Conclusions:**

These results suggest that FOXP3 variants could exert a role in the Treg, which could be one of the factors involved in the susceptibility and pathogenesis of IBD.

## Highlights

• FOXP3 variants are associated with susceptibility to IBD.
• GAGA haplotype is associated with reduced TGF-γ plasma levels in IBD.
• G/C haplotypes have a protective effect on susceptibility in CD.

## Introduction

Inflammatory Bowel Disease (IBD) is an immune-mediated chronic inflammatory disorder of the gastrointestinal tract. The two major clinical phenotypes of IBD are Ulcerative Colitis (UC) and Crohn's Disease (CD). Both diseases are associated with significant morbidity and have a major impact on an individual's quality of life.^1^ The exact etiology of IBD is not clearly understood, however, it is known that genetic predisposition and immunological imbalance have been implicated in the cause of this disorder.^2^, ^3^, ^4^

Genetics and immunology evidence has clarified that the innate and adaptive immune responses are equally important in inducing intestinal inflammation. Recently, the studied group demonstrated that Interleukin (IL) 6 genetic variants were associated with UC and CD susceptibility.^4^ About adaptive immune response, besides T-helper cell type (Th)1 and Th2 immune responses, other subsets of T-cells, namely Th17 and regulatory T (Treg) cells, are likely to play a role in IBD.^5^^,^^6^ Th2 cells and their cytokines are believed to be predominantly involved in UC pathogenesis, while Th1 cells are thought to play a major role in CD and Th17 cells are implicated in both conditions. On the other hand, Treg cells have important protective functions in the context of intestinal mucosal inflammation.^6^ Treg cells suppress effector T-cell responses by producing the pleiotropic cytokines Transforming Growth Factor (TGF)-β and IL-10, which are dominantly viewed as critical mediators for tolerance and immunosuppression.^7^^,^^8^

The Forkhead Box Protein 3 (FoxP3), a member of the transcription factor winged-helix family, is a crucial regulator of CD4^+^ CD25^+^ Treg cells development and function.^9^ The human FOXP3 gene is located on the short arm of chromosome X (Xp11.23) and variants in this gene may influence protein expression and function, reducing Treg cell activity and leading to autoimmunity development.^10^ The FOXP3-924 G>A (rs2232365) and -3279 C>A (rs3761548) are Single-Nucleotide Variants (SNV) in the promoter region, in which the A allele of both variants is associated with diminished FOXP3 expression.^10^^,^^11^ Furthermore, different SNVs in the promoter region of FOXP3 have been associated with the susceptibility and prognosis of autoimmune diseases, such as multiple sclerosis,^12^ rheumatoid arthritis,^13^ psoriasis,^14^ and systemic sclerosis.^15^

The studied group also demonstrated, in a recent study, the association of FOXP3 genetic variants with systemic lupus erythematosus susceptibility, disease activity, and TGF-β plasma levels.^16^ However, until now, few studies evaluated the FOXP3-924 G>A (rs2232365) and FOXP3-3279 C>A (rs3761548) variants in UC ^17^ and CD.^18^^,^^19^ In addition, there are no studies that evaluated TGF-β1 and IL-10 plasma levels according to these FOXP3 genotypes or haplotype structures. Moreover, there is no information about the association between those variants and score activity disease. Thus, this study aimed to evaluate the FOXP3-924 G>A (rs2232365) and FOXP3-3279 C>A (rs3761548) variants and their individual and haplotype associations with IBD susceptibility, clinical and endoscopic activity, as well as with TGF-β1 and IL-10 plasma levels in female patients with UC and CD.

## Subjects and methods

### Study subjects

As described previously in the group study, from July 2018 to May 2019, 110 female patients with IBD (UC = 60 and CD = 50), were consecutively recruited from the Gastroenterology Outpatient of the State University of Londrina, Londrina, Paraná, South Brazil.^4^ The diagnosis of IBD was established according to clinical evaluation and a combination of endoscopic, histological, radiological, and biochemical investigations as proposed in the 3^rd^ European Evidence-based Consensus on the Diagnosis and Management of UC ^20^ and CD.^21^ In addition, 154 female controls were selected among blood donors of the Regional Blood Bank of Londrina, from the same geographic region of the IBD patients. The exclusion criteria for all the individuals enrolled in the study were the presence of other autoimmune diseases, acute or chronic infectious diseases, heart, thyroid, kidney, hepatic, or oncologic diseases.^4^

Demographic, epidemiological, and anthropometric data (for patients and controls), as well as clinical history, symptoms, and treatment before the inclusion in this study (for patients) were obtained using a standard questionnaire at the admission of the individuals. Briefly, Body Mass Index (BMI) was calculated as weight (kg) divided by height (m) squared and the ethnicity was self-reported as Caucasian and non-Caucasian.

### Clinical and endoscopic examinations

In UC patients, the clinical activity was evaluated by the Partial Mayo Index Score and categorized as remission (< 2), mild activity (2‒4), moderate (5‒7), and severe (> 7).^4^^,^^22^ In CD patients, the clinical activity was assessed by Crohn's Disease Activity Index (CDAI) and the results were classified according to the activity status: remission (≤ 150), mild (151‒219), moderate (220‒450), and severe or very severe activity (> 450).^4^^,^^23^

The colonoscopies were performed by an experienced gastroenterologist with a Fujifilm endoscope (EC-250HL5-system EPX 2500 Fujinon, Minato-Ku, Tokyo, Japan), and immediately after the procedure, the findings were graded according to both Mayo endoscopic score for UC and Crohn's Disease Endoscopic Index of Severity (CDEIS) for CD.^4^ The inflammatory severity within the intestine determined by Mayo was classified in remission (0), mild activity (1), moderate (2), and severe (3) and, according to CDEIS values, as remission (< 3), mild (3‒8), moderate (9‒12), and severe (> 12).^4^^,^^24^ Based on their MAYO and CDEIS values, patients were divided into two groups of endoscopic activity: remission/mild (MAYO ≤ 1 or CDEIS ≤ 8) and moderate/severe (MAYO > 1 and CDEIS > 8) for statistical analysis.^4^^,^^21^

### Blood samples and immunological biomarkers

Venous blood samples, after fasting for 12h, were obtained with anticoagulant Ethylenediaminetetraacetic acid (EDTA), centrifuged at 3000 rpm for 15 min; further, plasma and buffy-coat were separated, divided into aliquots and stored at -80°C until thawed for assays, according to Flauzino et al.^12^ The IL-10 and TGF-β1 plasma levels were determined using microspheres multiplex immunofluorimetric assay (Procarta Plex High Sensitivity Assay by Thermo Fisher Scientific, Vienna, Austria) for the Luminex platform (MAGPIX™, Luminex Corp., Austin, TX, USA), that was performed according to the manufacturer's recommendations, as described in Stadlober et al.^16^

### DNA extraction

Genomic DNA was extracted from a buffy-coat of peripheral blood cells using a resin column procedure for FOXP3 genotyping, (PureLink Genomic DNA, Invitrogen by Life Technologies, Carlsbad, CA, USA), following the manufacturer's instructions.^4^^,^^16^ The DNA concentration was measured with a NanoDrop 2000c™ spectrophotometer (ThermoScientific, Waltman, MA, USA) at 260 nm and purity was assessed by measuring the 260/280 nm ratio.^16^

### FOXP3 genetic variants

Two SNV in the promoter region of the FOXP3 gene located on chromosome X were genotyped: -924 G>A (rs2232365) at position 49259426 and -3279 C>A (rs3761548) at position 49261784 according to listed in the international database and to GenBank accession number NG_007392.1, as described in Stadlober et al.^16^

Polymerase Chain Reaction (PCR) followed by Restriction Fragment Length Polymorphism (RFLP) analysis was used to detect the rs2232365 and rs3761548 SNV, as previously reported by Banin-Hirata et al.^25^ with some modifications described in Stadlober et al.^16^ For rs2232365 genotyping, the following primers were used: 5´-AGGAGAAGGAGTGGGCATTT-3´ (forward) and 5´-TGTGAGTGGAGGAGCTGAGG -3´ (reverse), according to Paradowska-Gorycka et al.^26^ The rs3761548 genotyping was performed with the following primers: 5′-GGCAGAGTTGAAATCCAAGC-3′ (forward) and 5’-CAACGTGTGAGAAGGCAGAA-3′ (reverse), according to He et al.^27^ As described in Stadlober et al.,^16^ all reactions were performed with negative control (without a DNA sample). The PCR products of rs2232365 [249 base pairs (bp)] were digested overnight at 37°C with Esp3I restriction endonuclease (Invitrogen™, Life Technologies, Carlsbad, CA, USA), generating two fragments of 132 bp and 117 bp corresponding to G allele, while the A allele that did not undergo enzymatic cleavage and remained with 249 bp. PCR products (155 bp) of rs3761548 were digested with PstI restriction endonuclease (Anza™, Invitrogen, Life Technologies, Carlsbad, CA, USA), which generated two fragments, 80 bp and 75 bp, that correspond to C allele, while the A allele remained with 155 bp. All PCR-RFLP products were analyzed using 10% polyacrylamide gel and stained with silver nitrate.^16^

### Statistical analysis

Analysis of contingency tables (χ2 or Fisher's exact test) was employed to evaluate the associations between categorical variables and diagnostic groups. The Kolmogorov-Smirnov test was used to assess the normality of distribution. TGF-β1 plasma levels were transformed into natural Logarithm (Ln) to ensure data normality. We assessed the differences in other continuous variables between groups using the Mann-Whitney test. The results were adjusted by extraneous variables using binary logistic regression. Categorical variables were expressed as absolute number (n) and percentage (%) and continuous variables were expressed as median and percentiles (25%‒75%).

Inference of recombination sites between FOXP3 alleles was determined using the PHASE software version 2.1.1 by assigning each haplotype with maximum probability.^28^^,^^29^ The estimation of pairwise linkage disequilibrium was performed in Haploview software version 4.2. by describing the D and r-squared value. The Hardy-Weinberg Equilibrium (HWE) was calculated. Binary or multinomial logistic regression analysis was performed to assess the effect of the genetic variants in the study group and related Odds Ratio (OR) and 95% Confidence Interval (CI) were determined. All statistical tests (p < 0.05) are considered as the significant level. Statistical analyses were performed using IBM SPSS windows version 24 (SPSS, Inc., Chicago, IL, USA).

## Results

### Characteristics of the subjects

IBD patients (UC+CD) and controls did not differ in ethnicity (p = 0.227) and smoking (p = 0.432). However, IBD patients were older [median = 50 years (38–56) vs. median = 36 years (28–44), p < 0.001] and had higher BMI [median = 26.6 kg/m^2^ (22.9–29.5) vs. 23.9 kg/m^2^ (21.7–27.1), p = 0.005] than controls (data not shown). Thus, the authors have adjusted the results for possible effects of age and BMI in multivariate analyses.

Table 1 shows the endoscopic and clinical characteristics of IBD patients according to the disease (UC and CD). Endoscopic activity evaluated by MAYO in UC patients demonstrated that 26 (43.3%) were in remission, 10 (16.7%) in mild, 17 (28.3%) in moderate, and 7 (11.7%) in severe activity. Clinical activity scores showed that 34 (56.7%) UC patients were in remission, 22 (36.7%) had mild, 3 (5.0%) moderate, and 1 (1.7%) severe activity. In CD patients, endoscopic activity evaluated by CDEIS demonstrated that the most of patients, 31 (62.0%) were in remission, while 8 (16.0%) had mild, 5 (10.0%) moderate, and 6 (12.0%) severe activity. According to clinical disease activity, 30 patients (60.0%) were in remission, 19 (38.0%) had mild, and 1 (2.0%) had moderate activity. None of the CD patients had severe or very severe clinical activity (CDAI > 450).

**Table 1:** Endoscopic and clinical parameters of female patients with Inflammatory Bowel Disease (IBD).

Characteristics	Ulcerative Colitis (n = 60)	Crohn Disease (n = 50)
Endoscopic Activity^a^		
Remission	26 (43.3)	31 (62.0)
Mild	10 (16.7)	8 (16.0)
Moderate	17 (28.3)	5 (10.0)
Severe	7 (11.7)	6 (12.0)
Clinical Activity^b^		
Remission	34 (56.7)	30 (60.0)
Mild	22 (36.7)	19 (38.0)
Moderate	3 (5.0)	1 (2.0)
Severe	1 (1.7)	0 (0.0)
Treatment		
Sulfasalazine or 5-aminisalicylic acid (Yes/No)	55 (91.7) / 5 (8.3)	9 (18.0) / 41 (82.0)
Corticosteroids (Yes/No)	8 (13.3) / 52 (86.7)	5 (10.0) / 45 (90.0)
TNF-α inhibitors (Yes/No)	11 (18.3) / 49 (81.7)	30 (60.0) / 20 (40.0)
Azathioprine or methotrexate (Yes/No)	17 (28.3) /43 (71.7)	34 (68.0) / 16 (32.0)

Data were expressed by absolute number and percentage. TNF, Tumor Necrosis Factor.

a Endoscopic activity in UC patients was evaluated by Mayo endoscopic score as remission (0), mild activity (1), moderate (2), and severe (3). In CD patients by Crohn's Disease Endoscopic Index of Severity (CDEIS) and classified in remission (< 3), mild (3‒8), moderate (9‒12), and severe (> 12).

b Clinical activity in UC patients was evaluated by Partial Mayo Index Score: remission (< 2), mild activity (2‒4), moderate (5‒7), and severe (> 7). In CD patients, clinical activity was assessed by Crohn's Disease Activity Index (CDAI) and the results were classified according to the activity status: remission (≤ 150), mild (151‒219), moderate (220‒450), and severe or very severe activity (> 450).

Regarding the IBD treatment, the patients were treated with a combination of medications. Most UC female patients, 55 (91.7%), were treated with aminosalicylates (Sulfasalazine or 5-aminosalicylic acid), 8 (13.3%) with corticosteroids, 11 (18.3%) with TNF-α inhibitors, and 17 (28.3%) with azathioprine or methotrexate. Most CD female patients, 34 (68.0%), were in treatment with azathioprine or methotrexate), 30 (60.0%) patients were treated with TNF-α inhibitors, 9 (18.0%) with aminosalicylates, and only 5 (10.0%) were in treatment with corticosteroids.

### FOXP3 genotype and IBD susceptibility

Table 2 shows the FOXP3-924 G>A (rs2232365) and FOXP3-3279 C>A (rs3761548) allelic and genotype frequency in different genetic models among patients with IBD and controls. A case-control analysis was performed to assess the influence of FOXP3 genetic variants on IBD susceptibility. The HWE was observed among the rs2232365 and rs3761548 genotype frequency (p > 0.05). However, the HWE was not observed in the rs3761548 in the control group (p = 0.034).

**Table 2:** Distribution of FOXP3-924 G>A (rs2232365) and FOXP3-3279 C>A (rs3761548) genotypes and allelic frequencies among Brazilian patients with Inflammatory Bowel Disease (IBD) and controls.

	Controls (n = 154)	IBD (n = 110)	UC (n = 60)	CD (n = 50)	Controls × IBD (p-value)*	Controls × UC (p-value)*	Controls × CD (p-value)*
rs2232365 -924 (G>A)							
Allele model							
G	170 (55.2)	120 (54.5)	70 (58.3)	50 (50.0)	Reference	Reference	Reference
A	138 (44.8)	100 (45.5)	50 (41.7)	50 (50.0)	0.214	0.892	0.056
Co-dominant model							
GG	44 (28.9)	29 (26.4)	18 (30.0)	11 (22.0)	Reference	Reference	Reference
GA	82 (53.9)	62 (56.4)	34 (56.7)	28 (56.0)	0.302	0.505	0.202
AA	26 (17.1)	19 (17.2)	8 (13.3)	11 (22.0)	0.193	0.914	**0.047**^a^
Dominant model							
GG	44 (28.9)	29 (26.4)	18 (30.0)	11 (22.0)	Reference	Reference	Reference
GA+AA	108 (71.1)	81 (73.6)	42 (70.0)	39 (78.0)	0.219	0.610	0.105
Recessive model							
GG+GA	126 (82.9)	91 (82.7)	52 (86.7)	39 (78.0)	Reference	Reference	Reference
AA	26 (17.1)	19 (17.2)	8 (13.3)	11 (22.0)	0.368	0.627	0.097
**rs3761548 -3279 (C>A)**							
C	215 (69.8)	140 (63.6)	73 (60.8)	67 (67.0)	Reference	Reference	Reference
A	93 (30.2)	80 (36.4)	47 (39.2)	33 (33.0)	0.478	0.287	0.896
Co-dominant							
CC	69 (44.8)	45 (40.9)	24 (40.0)	21 (42.0)	Reference	Reference	Reference
CA	77 (50.0)	50 (45.5)	25 (41.7)	25 (50.0)	0.794	0.800	0.962
AA	8 (5.2)	15 (13.6)	11 (18.3)	4 (8.0)	0.141	0.053	0.510
Dominant model							
CC	69 (44.8)	45 (40.9)	24 (40.0)	21 (42.0)	Reference	Reference	Reference
CA+AA	85 (55.2)	65 (59.1)	36 (60.0)	29 (58.0)	0.891	0.779	0.934
Recessive model							
CC+CA	146 (94.8)	95 (86.4)	49 (81.7)	46 (92.0)	Reference	Reference	Reference
AA	8 (5.2)	15 (13.6)	11 (18.3)	4 (8.0)	0.104	**0.041**^b^	0.482

The genotyping was successful in 110 cases and 152 controls for rs2232365 and 110 cases and 154 controls for rs3761548. Bold values represent statistically significant values; IBD, Inflammatory Bowel Disease; UC, Ulcerative Colitis; CD, Crohn's Disease; OR, Odds Ratio; 95% CI, Confidence Interval.

⁎ p-value adjusted by age, ethnicity, body mass index, and smoking.

a OR = 3.147, 95% CI 1.015–9.758, p = 0.047.

b OR = 3.221, 95% CI 1.050–9.876, p = 0.041.

The FOXP3-924 G>A rs2232365 did not differ in patients with IBD and UC in comparison to controls. In CD patients, the presence of AA genotype in the codominant model was higher compared to controls, 11 (22.0%) vs. 26 (17.1%), respectively (OR = 3.147, 95% CI 1.015–9.758, p = 0.047). Furthermore, there was a statistical trend toward an association between the presence of the A allele of rs2232365 and CD (p = 0.056). Regarding FOXP3-3279 C>A (rs3761548), the frequency of the genotypes did not differ in patients with IBD and CD in comparison to controls. In UC patients, the presence of AA genotype in the recessive model was higher compared to controls, 11 (18.3%) vs. 8 (5.2%), respectively (OR = 3.221, 95% CI 1.050–9.876), p = 0.041), also, there was a statistic trend toward an association of the presence of AA genotype in the codominant model and UC (p = 0.053). All the results were adjusted by age, ethnicity, BMI, and smoking.

### FOXP3 haplotype structures and IBD susceptibility

Four possible haplotype combinations from rs2232365 and rs3761548 were investigated in the present study: A/C, A/A, G/C, and G/A. The linkage disequilibrium between FOXP3 rs2232365 and rs3761548 showed that those SNVs are not good surrogate markers for each other due to the low correlation coefficient presented (D′ = 0.983; r^2^ = 0.386); therefore, it is important to assess their combined effects.

In the association study of FOXP3 haplotypes, the following models were analyzed: A/C dominant (A/C carriers vs. A/A, G/C, and G/A carriers), A/C recessive (ACAC vs. A/A, G/C, and G/A carriers), G/C dominant (G/C carriers vs. A/C, A/A, and G/A carriers), G/C recessive (GCGC vs. A/C, A/A, and G/A carriers), G/A dominant (G/A carriers vs. A/C, A/A, and G/C carriers), and G/A recessive (GAGA vs. A/C, A/A, and G/C carriers). The A/A combination and the G/C recessive haplotype were rare (< 5.0%) and were excluded from the analysis. The predominant haplotype was A/C while the less frequent haplotype was A/A in the patient cohort.

Table 3 shows the distribution of FOXP3-924 G>A (rs2232365) and FOXP3-3279 C>A (rs3761548) haplotypes among patients with IBD and controls from the Brazilian population. The authors found an association between the G/A recessive haplotype with IBD (OR = 4.003, 95% CI 1.100–14.56, p = 0.035) and UC (OR = 6.107, 95% CI 1.609–23.18, p = 0.008) adjusted by age, sex, ethnicity, BMI, and smoking. However, this association was not found in CD patients (OR = 2.215, 95% CI 0.303–16.20, p = 0.433). On the other hand, the authors found a protective effect of the G/C haplotype in the dominant model with CD patients (OR = 0.432, 95% CI 0.196–0.951, p = 0.037) adjusted by age, sex, ethnicity, BMI, and smoking, but not with IBD (p = 0.088) and UC (p = 0.494).

**Table 3:** Distribution of rs2232365 -924 (G>A) and rs3761548 -3279 (C>A) FOXP3 haplotypes among Brazilian patients with Inflammatory Bowel Disease (IBD) and controls.

Haplotypes (rs2232365/ rs3761548)	Controls, n (%)	IBD, n (%)	OR (95% CI)	p^a^	UC, n (%)	OR (95% CI)	p^a^	CD, n (%)	OR (95% CI)	p^a^
A/C dominant	110 (72.8)	80 (72.7)	1.270 (0.679 – 2.377)	0.454	42 (70.)	1.038 (0.499 – 2.160)	0.920	38 (76.0)	1.628 (0.692 – 3.825)	0.264
A/C recessive	28 (18.5)	19 (17.3)	1.275 (0.622 – 2.615)	0.507	8 (13.3)	0.764 (0.304 – 1.921)	0.764	11 (22.0)	1.934 (0.780 – 4.792)	0.154
G/A dominant	82 (54.3)	65 (59.1)	1.077 (0.614 – 1.890)	0.796	36 (60.0)	1.163 (0.598 – 2.262)	0.656	29 (58.0)	1.065 (0.505 – 2.247)	0.869
G/A recessive	5 (3.3)	14 (12.7)	4.003 (1.100 – 14.56)	**0.035**	11 (18.3)	6.107 (1.609 – 23.18)	**0.008**	3 (6.0)	2.215 (0.303 – 16.20)	0.433
G/C dominant	67 (44.4)	39 (35.5)	0.606 (0.341 – 1.077)	0.088	22 (36.7)	0.789 (0.400 – 1.555)	0.494	17 (34.0)	0.432 (0.196 – 0.951)	**0.037**

Bold values represent statistically significant values; OR, Odds Ratio; 95% CI, Confidence Interval; IBD, Inflammatory Bowel Disease; UC, Ulcerative Colitis; CD, Crohn's Disease.

a p-value adjusted by age, ethnicity, body mass index, and smoking. Haplotype models: A/C dominant (A/C carriers vs. A/A, G/C, and G/A carriers), A/C recessive (ACAC vs. A/A, G/C, and G/A carriers), G/A dominant (G/A carriers vs. A/C, A/A, and G/C carriers), G/A recessive (GAGA vs. A/C, A/A, and G/C carriers), and G/C dominant (G/C carriers vs. A/C, A/A, and G/A carriers).

### Cytokines plasma levels and FOXP3 genetic variants

**Fig. 1:**
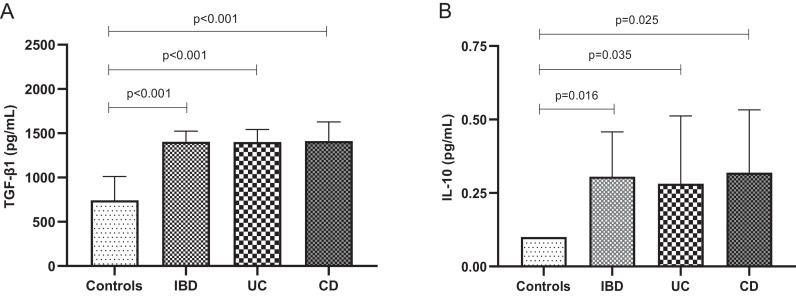
Cytokine plasma levels in patients with Inflammatory Bowel Disease (IBD), Ulcerative Colitis (UC), Crohn's Disease (CD) and controls. A) Transforming Gowth Factor beta 1 (TGF-β1) plasma levels in IBD, UC and CD patients compared to controls; B) Interleukin 10 (IL-10) plasma levels in IBD, UC and CD patients compared to controls; results were expressed as median and confidence interval 95%; p-value adjusted by age, ethnicity, body mass index, smoking, and treatment.

IBD, as well, as UC and CD patients showed higher levels of TGF-β1 and IL-10 than controls (Fig. 1). All results in Fig. 1 were adjusted by age, ethnicity, BMI, and smoking. However, TGF-β1 and IL-10 plasma levels did not differ according to FOXP3-924 G>A (rs2232365) and -3279 C>A (rs3761548) genotypes (dominant and recessive models) in IBD, as well as among the UC and CD patients, and controls (data not shown).

**Fig. 2:**
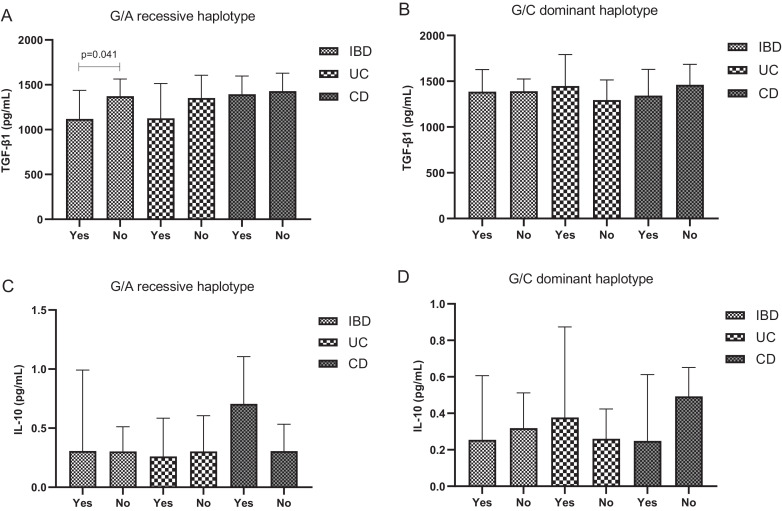
Cytokine plasma levels according to the haplotypes of FOXP3-924 A>G (rs2232365) and FOXP3-3279 C>A (rs3761548) variants in Inflammatory Bowel Disease (IBD) patients. A) Transforming Growth Factor beta 1 (TGF-β1) plasma levels in G/A recessive haplotype in IBD, Ulcerative Colitis (UC), and Crohn's Disease (CD) female patients; B) TGF-β1 plasma levels in G/C dominant haplotype in IBD, UC and CD female patients; C) Interleukin 10 (IL-10) plasma levels in G/A recessive haplotype in IBD, UC, and CD female patients; D) IL-10 plasma levels in G/C dominant haplotype model in IBD, UC, and CD female patients; results expressed as median and 95% confidence interval; p-value adjusted by age, ethnicity, body mass index, smoking, and treatment. Haplotype models: G/A recessive (GAGA vs. A/C, A/A, and G/C carriers) and G/C dominant (G/C carriers vs. A/C, A/A, and G/A carriers). Yes, presence of haplotype; No, other haplotypes

The plasma levels of TGF-β1 and IL-10 according to FOXP3-924 G>A and FOXP3-3279 C>A haplotype structures in IBD patients are shown in Fig. 2. IBD patients with the GAGA haplotype (G/A recessive haplotype) had lower TGF-β1 plasma levels (p = 0.041) than other haplotypes (A/C, A/A, or G/C carriers), after adjusted by age, ethnicity, BMI, and smoking. However, TGF-β1 plasma levels did not differ when the authors analyzed UC and CD separately (Fig. 2A). TGF-β1 plasma levels also did not differ in IBD, UC, and CD according to the G/C dominant haplotype model (Fig. 2B). In addition, IL-10 plasma levels also did not differ according to FOXP3 haplotype models in IBD, as well as among the patients with UC and CD (Fig. 2C and 2D, respectively). In the control group, cytokine plasma levels did not differ according to FOXP3 haplotype structures (data not shown).

### FOXP3 genetic variants and IBD clinical and endoscopic activity

**Fig. 3:**
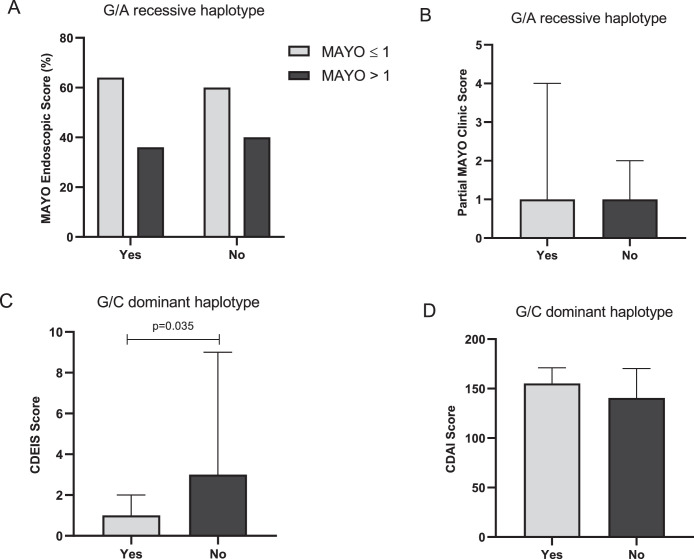
Endoscopic and clinical activity according to the haplotypes of FOXP3-924 A>G (rs2232365) and FOXP3-3279 C>A (rs3761548) variants in Inflammatory Bowel Disease (IBD) patients. A) Endoscopic activity in Ulcerative Colitis (UC) patients was evaluated by Mayo endoscopic score in G/A recessive haplotype model; B) Clinical activity in UC patients was evaluated by Partial Mayo Index Score G/A recessive haplotype model; C) Endoscopic activity in Crohn's Disease (CD) patients was evaluated by Crohn's disease endoscopic index of severity (CDEIS) in G/C dominant haplotype model; D) Clinical activity in CD patients was evaluated by Crohn's Disease Activity Index (CDAI) in G/C dominant haplotype model. Haplotype models: G/A recessive (GAGA vs. A/C, A/A, and G/C carriers) and G/C dominant (G/C carriers vs. A/C, A/A, and G/A carriers). Yes, Presence of haplotype; No, Other haplotypes.

Regarding endoscopic and clinical activity, the authors did not find an association between the FOXP3-924 G>A and FOXP3-3279 C>A genotypes (dominant and recessive models) and disease activity in UC and CD patients (data not shown). Moreover, when the authors analyzed those variants in haplotypes, no significant association was found between GAGA haplotype and Mayo endoscopic and partial Mayo score in UC patients (Fig. 3A and 3B, respectively). However, the authors found that CD patients with G/C haplotype (dominant model) had diminished CDEIS scores (p = 0.035, Fig. 3C) and a higher frequency of patients (p = 0.046) in remission/mild activity (CDEIS < 8) (data not shown). In addition, G/C haplotype structure (dominant model) was not associated with clinical activity evaluated by CDAI (Fig. 3D).

## Discussion

The main findings of the present study were that the AA genotype of FOXP3-924 G>A (rs2232365) was associated with a 3-fold chance of developing CD than other genotypes, while the AA genotype of FOXP3-3279 C>A (rs3761548) was associated with a 3-fold chance of developing UC than other genotypes. However, FOXP3 variant genotypes (dominant and recessive models) were not associated with TGF-β1 and IL-10 plasma levels as well as endoscopic/clinical activity disease scores. In addition, the authors found an association between the GAGA haplotype with IBD and UC, but not with CD. IBD patients with the GAGA haplotype had a 6-fold higher chance to develop UC and lower TGF-β1 plasma levels than other haplotype structures. Moreover, the G/C haplotype (dominant model) showed a protective effect of 60.0% in the susceptibility to CD and lower CDEIS endoscopic score than other haplotypes.

In most patients with IBD, a large number of common genetic variants (> 1% allelic frequency in the general population) have been associated with the disease susceptibility in a polygenic setting.^30^ The previous study ^31^ has also led to the confirmation of more than 163 genetic loci containing susceptibility genes for IBD. However, UC and CD display heterogeneity in inflammatory and symptomatic burden between and within individuals over time, and the association of genetic variants with the disease activity and severity has been less investigated and the successful identification of genetic variants associated with complex diseases, such as CD and UC, has raised the exciting possibility of a more personalized approach to clinical management. In IBD, this quest is particularly urgent because of the substantial heterogeneity in the disease course, and individual response to therapy.

Previous studies have already shown the association between certain genetic markers and the prediction of more aggressive diseases and the necessity of surgery.^32^ As for UC, the HLA DRB1*0103 allele is associated with pancolitis and the need for colectomy.^33^^,^^34^ In a large Dutch CD cohort study, Weersma et al.^35^ showed an increased number of risk alleles at 5 risk loci (NOD2, IBD5, DLG5, ATG16L1, and IL23R) is associated with a more severe disease course. The IBD CHIP project ^36^ concluded that carriage of some NOD2 variants is an independent predictive factor for ileal CD, structuring and penetrating behaviors, and the need for surgery. Other genetic markers including PRDM1 variants, IL23R, JAK2, and TNFS15 also appear to be associated with progressive, severe CD.^37^

Regarding the FOXP3 genetic variants evaluated in the present study, FOXP3 is the most specific and reliable biomarker of Treg cells and is essential for driving CD4^+^ CD25^+^ FOXP3^+^ Treg cell function.^38^ Previous studies demonstrated that variations in FOXP3 may cause immune response impairment and contribute to X-linked autoimmune disease development.^12^^,^^14^^,^^15^^,^^39^ The association between the FOXP3-924 G>A (rs2232365) variant with CD susceptibility in the present study suggests a functional defect on Treg in patients with the AA genotype. The G>A substitution is located in a putative-binding site for the transcription factor GATA-3.^40^ This transcription factor binds to the promoter region of FOXP3 to inhibit its expression only when the A allele is present. To occur FOXP3 expression, GATA-3 must be removed from the promoter region.^41^ So, GG carriers lose their GATA-3-binding site, enabling FOXP3 gene transcription. Previous studies failed to find any association of FOXP3-924 G>A with CD susceptibility in female patients.^18^^,^^19^ The inconsistent result observed in this previously reported study may be also explained by differences in the cohort ethnicity.

The present data also demonstrated that the AA genotype of the FOXP3-3279 C>A (rs3761548) variant confers 3-fold more chance of developing UC. This result could suggest a functional defect in Treg in patients with the AA genotype. Similarly, another study found an association between the FOXP3-3279 C>A variant and UC in the Chinese population.^17^ Also, these authors showed that female patients with the AA genotype have decreased expression of FOXP3 mRNA and protein compared to the CC genotype. The presence of the A allele alters the promoter region and consequently, there is a loss of binding of some transcription factors, such as E47 and C-Myb, leading to defective transcription of FOXP3,^10^ and therefore, might affect the function or quantity of Tregs.^42^ Regarding CD female patients, the authors did not find an association between the FOXP3-3279 C>A variant and CD susceptibility. The present results are in agreement with previous studies that also did not demonstrate this association in CD female patients.^18^^,^^19^

Genetic variants do not exert great influence by themselves ^43^ and haplotype analysis could be better to understand the association between genetic variants and disease susceptibility. Thus, the authors investigated the haplotype structures of FOXP3-924 G>A (rs2232365) and -3279 C>A (rs3761548) variants and demonstrated an association between the G/A haplotype in the recessive model (GAGA carriers), with IBD and UC patients. Furthermore, the authors found that the susceptibility to UC in haplotype was 2-fold higher than in genotype analysis. On the other hand, the G/C haplotype (dominant model) showed a protective effect on CD female patients.

In addition, the authors found higher TGF-β1 and IL-10 plasma levels in patients with IBD, UC, and CD than in the control group. The present data are in agreement with previous studies that also demonstrated higher TGF-β1 plasma levels in UC ^44^ and CD.^45^ Moreover, Kiliç et al.^46^ suggested that TGF-β1 could be used as a marker for differential diagnosis of active UC. TGF-β1 has several important functions in the pathogenesis of IBD that include control of epithelial cells proliferation and differentiation, immunosuppression, and regulation of extracellular matrix formation. TGF-β1 inhibits proliferation and stimulates differentiation of epithelial cells that aids in the repair process of the mucosa following the damage.^47^

IL-10 is an anti-inflammatory cytokine produced by Treg, macrophages M2 and other cells to modulate the inflammatory response.^7^ The higher IL-10 plasma levels demonstrated in the present study in IBD patients (UC and CD) than controls are also in agreement with other studies carried out in UC patients ^45^^,^^48^, ^49^, ^50^ but not in CD patients.^45^^,^^48^^,^^50^

IL-10 acts directly on Treg cells to maintain FOXP3 expression and their suppressive capacity,^51^ as well as on antigen-presenting cells.^52^ Moreover, infliximab treatment (a TNF-α inhibitor) could increase TGF-β1 and IL-10 serum levels in CD patients.^53^ In this study, 60.0% of CD patients used TNF-α inhibitors in combination with other therapies. In the treatment of both CD and UC, combination therapy is recommended because it is more effective than monotherapy, in which each drug is used separately.^54^ Thus, the present data suggested that elevated TGF-β1 and IL-10 plasma levels could be related to inflammation activity and therapy response and could represent compensatory mechanisms involved in mucosal healing.

Furthermore, in the present study, the FOXP3 variants evaluated could alter the TGF-β1 and IL-10 levels. IBD patients with GAGA haplotype (recessive model) showed diminished TGF-β1 plasma levels, but not IL-10, compared to other haplotype structures. However, when the authors evaluated the FOXP3-924 G>A and -3279 C>A genotypes individually, in the dominant and recessive models, the authors did not find an association between them and the plasma levels of TGF-β1 and IL-10. The present data suggested that the GAGA haplotype could interfere with the Treg cell function by decreasing FOXP3 expression, and consequently decreasing the TGF-β1 levels. Studies support the role of TGF-β1 as a negative regulator of mucosal inflammation and indicate that defective production/activity of this cytokine can lead to the development of or exacerbate colitis.^55^^,^^56^ The authors did not find an association between FOXP3 genetic variants and IL-10 plasma levels, which could be explained by the fact of many other cell types can produce this cytokine and IL-10 plasma levels can be affected by genetic variants in IL10.^57^, ^58^, ^59^

Regarding endoscopic and clinical disease activity, the G/C dominant haplotype model was associated with diminished endoscopic activity evaluated by CDEIS. However, when the authors evaluated the FOXP3-924 G>A (rs2232365) and FOXP3-3279 C>A (rs3761548) genotypes individually, in the dominant and recessive models, the authors did not find an association between them and endoscopic/clinical disease activity scores. The authors hypothesize that the benefic effect of the G/C dominant haplotype model in CD development and endoscopic activity can be due to the presence of the G allele of FOXP3-924 G>A and the A allele of FOXP3-3279 C>A, as both were associated with higher FOXP3 expression. Furthermore, the complex interaction between FOXP3 haplotype structures/genotypes and plasma levels of TGF-β1 and IL-10, as well as endoscopic/clinical disease activity scores in IBD female patients, deserves further investigation.

Some limitations of this study should be considered. First, this is a case-control design, which does not allow inferences of a causal relationship; second, the ethnicity of patients and controls was self-reported; third, the authors evaluate the FOXP3 variants only in females; and fourth, the detection of the circulating IL-10 and TGF-β1 instead of the cytokine expression in the gut. However, previous studies showed high both serum and mucosal IL-10 levels in patients with active IBD.^60^^,^^61^ Actually, due to the essentially local effects of cytokines, the study of their circulating levels may not represent the concentration of these cytokines locally produced in the site of inflammation and is of limited value for a global understanding of the pathophysiology of these mediators. Moreover, the detectable serum levels of IL-10 and TGF-β does not take into account the membrane-bound form of these cytokines and the cytokine mRNA detection on tissue fragments may allow the identification of the cytokines directly involved in IBD. However, this method is very sensitive, but time-consuming and needs to use biological material which is not easily accessible for this purpose. Therefore, the authors intend to evaluate the potential clinical use of the FOXP3 genetic variants and the serum levels of the IL-10 and TGF-β as surrogate diagnostic biomarkers of IBD susceptibility, and clinical and endoscopy activity.

However, this study has some strengths, such as the adjusted results for many confounding variables and the determination of the genotype and haplotype frequencies, one reasonable explanation for the evaluation of the FOXP3 genetic variants only in female individuals. The authors decided to study only female patients with CD and UC considering that the FOXP3 is positioned on the X chromosome and the determination of the genotype frequencies, as well as haplotype frequencies, could be possible only in female individuals. In male individuals, the authors can determine only the allele frequency. Therefore, the statistical analysis and the discussion of the results must be separated due to the absence of the homozygous variant genotype (AA) in male individuals. In other previous studies, the authors studied the FOXP3 variant in male and female patients with multiple sclerosis and the authors divided the results into both groups and discussed them separately. The analysis and discussion of the results must be separately taken into account the absence of the homozygous variant genotype in male individuals. Therefore, the inclusion of only female individuals in the present study may not impair the results obtained. Another important and reasonable question is whether the presence of the AA genotype prevents the fixation of certain transcription factors by inhibiting the transcription of FOXP3.^10^ These authors showed that resting CD4^+^CD25^+^ T-cells from patients with the AA genotype had lower FOXP3 transcription/expression levels than those from patients with the CC genotype *in vitro* for transcription and translation levels comparison.

In conclusion, this is the first study to demonstrate the association between FOXP3 variants (rs2232365 and rs3761548) in female Brazilian patients with IBD, and these results suggest that FOXP3 variants, individually or in haplotype structures, could exert a role in the Treg cell function, which could be one of the factors involved in the susceptibility and pathogenesis of IBD in females.

## Authors’ contributions

Study Design: Andréa Simão, Edna Reiche, Marcell Lozovoy, Lucilene Westmore.

Data Collection and Experiments: Claudia Inoue, Tamires Flauzino, Beatriz Piantoni, Jaqueline Castardo, Talita Galvão, Paula Kikuchi, Camila Cataldi.

Statistical Analysis: Tamires Flauzino, Andréa Simão, Edna Reiche.

Data Interpretation: Claudia Inoue, Tamires Flauzino Marcell Lozovoy, Andréa Simão.

Manuscript Preparation: Claudia Inoue, Tamires Faluzino, Andréa Simão.

Literature Search: Claudia Inoue, Marcell Lozovoy, Edna Reiche, Andréa Simão, Lucilene Westmore.

## Funding

The study was supported by grants from the Coordination for the Improvement of Higher Level of Education Personnel (CAPES) and the Institutional Program for Scientific Initiation Scholarship (PIBIC) of The National Council for Scientific and Technological Development (CNPq).

## Ethical approval

This study was conducted after approval by the Institutional Research Ethics Committees of the University of Londrina, Paraná, Brazil (CAAE: 91833018.2.0000.5231). All procedures performed in studies involving human participants were in accordance with the ethical standards of the institutional and/or national research committee and with the 1964 Helsinki declaration and its later amendments or comparable ethical standards.

## Consent to participate

All the participants included in this study were informed in detail about the research and gave written informed consent.

## Conflicts of interest

The authors declare no conflicts of interest.
